# Experimental Investigations into the Action and Uses of the Bromide of Potassium

**Published:** 1866-02

**Authors:** 


Experimental Investigations in to the Actionand Uses of the Bromide of Potassium. By Roberts Bartholow, M.D., Prof. of Physics and Medical Chemistry in the Medical College of Ohio.

This is a little pamphlet of 16 pages, the contents of which were first published in the *Cincinnati Lancet and Observer.* The detail of the author's experiments with the bromide of potassium are interesting and instructive. The following are the conclusions at which he arrives in relation to the *modus operandi* and therapeutic action of the drug:—

After a careful survey of all the facts we are able to construct a theory of the action and *methodus medendi* of the bromide of potassium. I put this in the shape of the following conclusions:—

1. The bromide of potassium acts by absorption into the blood;

2. Its effects are expended upon the nervous centres, or the cerebro spinal axis;

3. Sedation of the heart and circulation, and the various local sedative effects are secondary results of the impression made upon the nervous centres;

4. Its physiological effects are not very decided, and are easily modified by any local disturbance;

5. Its therapeutical action is still more decidedly influenced by local morbid processes;

6. It is indicated where a sedative to the nervous system is required—in insomnia; too great reflex excitability; nervous and spasmodic affections of the larynx and bronchi; sexual excitement; and in an irritable state of the sexual organs;

7. It will be effectual in the foregoing conditions, in proportion to the degree in which structural lesions are absent, or in other words, in proportion to the degree in which these morbid states are functional rather than organic.

Dr. Bartholow thinks the bromide of potassium less efficient as an alterative than the iodide.

